# Patient satisfaction with conventional complete denture prosthesis: A cross-sectional study at Penang international dental college in Penang, Malaysia

**DOI:** 10.6026/973206300212332

**Published:** 2025-08-31

**Authors:** Priyanka Tiwari, Vethavalle Raveechandran, Varschini A.P Vasu, Kavithaanjali A.P Thanabal, Jayashri Tamanna Nerali

**Affiliations:** 1 Department of Prosthetic Dentistry, Penang International Dental College, Butterworth, Penang, Malaysia; 2 Department of Family Dentistry, Penang International Dental College, Butterworth, Penang, Malaysia

**Keywords:** Complete denture, patient satisfaction, prosthodontics

## Abstract

The patient satisfaction followed by receiving of complete dentures at the Penang International Dental College, Prosthetic Dentistry Department. A cross-sectional analysis was conducted using a 15-item questionnaire to assess the experiences of 100 patients. Data indicated high overall satisfaction, particularly with maxillary dentures, and no significant relationship was found between satisfaction and factors such as previous denture use or gender. Patients were generally content with the operator's professionalism and performance. Thus, we show that complete dentures at Penang International Dental College meet patients' expectations regarding satisfaction with their dentures.

## Background:

Edentulism, the loss of all natural teeth, affects both oral function and psychological well-being, with global incidence ranging from 7% to 69% [1]. It significantly impacts the quality of life (QOL) of older people, particularly in terms of eating, speaking and social interactions [2]. Conventional complete dentures are the most common, affordable, and easiest to maintain solution for restoring edentulous jaws. However, the success of complete dentures depends on patient satisfaction, which is influenced by factors such as fabrication technique, patient-related aspects and dentist-related factors [3]. In prosthodontic treatment, patient satisfaction is crucial for success and various factors, including the quality of dentures, oral health, personality and patient-dentist interactions, play a role [4]. Patient satisfaction is influenced not only by biological and technical aspects but also by psychological variables, age, gender, social circumstances, and marital status [5, 6]. Therefore, it is of interest to assess patient satisfaction with complete dentures at Penang International Dental College, focusing on patient-related and dentist-related factors using a questionnaire.

## Methodology:

This study employed convenience sampling, involving 100 patients who registered for complete dentures at the Prosthetic Department of Penang International Dental College between September 2022 and December 2023. The study focused on patients from Batch 24 and the Class of 2023 and received approval from the Penang International Dental College ethical review committee. The same clinician performed all procedures. The inclusion criteria were patients aged 50 to 80 with edentulous maxillary and mandibular jaws, who could understand and respond to English. Patients with temporomandibular disorders, neurological or psychological conditions, or a history of jaw surgery due to cancer or trauma were excluded. Dentures were fabricated by 4th- and 5th-year students under the supervision of faculty staff in the Prosthodontic department, following standardized procedures. These included obtaining patient consent and medical history, taking preliminary impressions with alginate, creating special trays, performing border moulding, and fabricating the denture base with self-cure acrylic resin. Jaw relations were recorded, followed by try-in, teeth setting, and polymerization. The Mann-Whitney test was used to assess the association between patient satisfaction levels, gender, and previous experience. Descriptive statistics (SPSS 26.0) were employed to explore relationships between variables and provide basic information. This research helps Penang International Dental College understand how patients respond to care in the Prosthetics Department, allowing for the correction of any errors. Additionally, it helps the doctor identify treatment-related issues and address them with future patients. The Questionnaire of the study is shown in Table 1 (see PDF). The descriptive analysis of satisfaction items is shown in Table 3 (see PDF), which presents the mean and standard deviation for various satisfaction items. Table 4 (see PDF) illustrates the correlation between patient satisfaction and independent variables, including gender and prior denture use experience.

## Results:

This study employed convenience sampling, involving 100 patients who registered for complete dentures at the Prosthetic Department of Penang International Dental College between September 2022 and December 2023. The study focused on patients from Batch 24 and the Class of 2023 and received approval from the Penang International Dental College ethical review committee. The same clinician performed all procedures. The inclusion criteria were patients aged 50 to 80 with edentulous maxillary and mandibular jaws, who could understand and respond to English. Patients with temporomandibular disorders, neurological or psychological conditions, or a history of jaw surgery due to cancer or trauma were excluded. Dentures were fabricated by 4th- and 5th-year students under faculty supervision following standardized procedures. These included obtaining patient consent and medical history, taking preliminary impressions with alginate, creating special trays, performing border moulding, and fabricating the denture base with self-cure acrylic resin. Jaw relations were recorded, followed by try-in, teeth setting, and polymerization. After denture insertion, patients received instructions on care and a follow-up visit was scheduled for one week later. The Mann-Whitney test was used to assess the association between patient satisfaction levels, gender, and previous experience. Descriptive statistics (SPSS 26.0) were employed to explore relationships between variables and provide basic information. This research helps Penang International Dental College understand how patients respond to care in the Prosthetics Department, allowing for the correction of any errors. Additionally, it helps the doctor identify treatment-related issues and address them with future patients. The socio-demographic characteristics of the participants are presented in Table 2 (see PDF). Of the 100 participants, 51% were male (n = 51) and 49% were female (n = 49). Regarding age, 47% of the participants were between the ages of 61-70, 33% were between 50-60, and 20% were aged 71 and above. In terms of prior denture experience, 66% had used dentures previously, and 34% had no prior denture experience. For the evaluation of previous denture use, 59.09% rated it as average, 15.15% as fair, 16.67% as good, and 9.09% as excellent, while no participants rated their past experience as poor.

Table 3 (see PDF) provides a descriptive analysis of patient satisfaction with their conventional removable complete dentures. The patients reported high satisfaction levels in various aspects of denture quality. The aesthetics of the denture base received a mean score of 4.3 (SD = 0.674), indicating good satisfaction with the appearance. The aesthetics of the artificial teeth scored slightly higher, with a mean of 4.34 (SD = 0.685), and facial aesthetics (lip and cheek support) scored 4.26 (SD = 0.648). Regarding functionality, the denture's ability to restore function had a mean score of 4.17 (SD = 0.726), while the ability to restore speech scored 4.28 (SD = 0.78). The satisfaction with maxillary denture retention during function and speech was both rated at 4.22 (SD = 0.824), while mandibular denture retention during function was slightly higher at 4.23 (SD = 0.777). Mandibular denture retention during speech had a mean score of 4.19 (SD = 0.813). The highest-rated item was the operator's demeanor and performance, with a mean score of 4.59 (SD = 0.605). The overall satisfaction level was reported as 5.57, with a standard deviation of 7.356, indicating a range of satisfaction across the patient population. Table 4 (see PDF) presents the results of the Mann-Whitney test to assess the correlation between patient satisfaction and independent variables such as gender and prior denture use experience. The analysis found no significant difference in satisfaction based on gender. The mean rank for male participants was 46.18 (n = 49), and for female participants, it was 54.65 (n = 51), with a Z-value of -1.41 and a p-value of 0.158. Since the p-value was greater than 0.05, the difference in satisfaction between genders was not statistically significant. Similarly, no significant difference in satisfaction was found between those with prior denture use experience (mean rank = 52.61, n = 66) and those without (mean rank = 52.61, n = 34), with a Z-value of -1.02 and a p-value of 0.309. The results from this study suggest that patients generally reported high satisfaction with their conventional removable complete dentures, particularly with the aesthetics and functional aspects such as retention and speech. The satisfaction levels were consistent across genders and prior denture experience, with no significant differences found based on these variables. These findings indicate that while patient satisfaction is influenced by the quality and fit of the dentures, factors such as gender and previous experience with dentures did not significantly impact overall satisfaction. The data from this study provide useful insights for the Prosthodontic department, helping to address potential areas of improvement and enhance patient care in future treatments.

## Discussion:

This study evaluated patient satisfaction with conventional removable complete dentures, revealing high levels of satisfaction, particularly regarding aesthetics and denture function. Interestingly, no significant differences in satisfaction were observed based on gender or prior denture experience, which contrasts with findings from some earlier studies that suggested these factors play a role in satisfaction. Razdan *et al.* (2025) [7] found that prior denture experience tends to improve satisfaction, particularly in terms of fit and functionality. A point echoed by Murrell GA (1988) [8], who noted that aesthetic concerns were crucial for patients with edentulism. Salim (2023) [9] stressed the significance of effective practitioner-patient interactions. Furthermore, Janto *et al.* (2022) found that although some patients initially struggled with fit and aesthetic concerns, satisfaction improved over time [10]. In terms of denture retention, Soboleva *et al.* (2022) [3] study found greater satisfaction with maxillary dentures due to better retention. This supports the idea that the larger surface area available for maxillary denture retention provides more stability compared to mandibular dentures, which tend to have smaller bearing surfaces and often lead to mandbular satisfaction. Nonetheless, the study highlights the importance of managing patient expectations, ensuring proper clinical supervision, and allowing sufficient time for adaptation to maximize satisfaction with removable dentures.

Several recommendations based on the information gathered and examined. Firstly, suggested scheduling follow-up appointments at one, two, and four weeks after denture placement to allow for further modifications that ensure maximum comfort for the patient. Additionally, conduct a study that compares patient expectations with satisfaction regarding complete dentures, both before and after the therapy. Lastly, for the convenience of patients, it is advised that the questionnaire be made available in multiple languages, including Malay, Mandarin, and Tamil, as these are the common languages used in Malaysia. Some limitations in the study were found. Firstly, the inclusion of both prosthodontists and students in this study may have influenced the findings, as students were limited by the number of dentures they could provide during their studies. Additionally, the fact that only one follow-up was conducts due to scheduling constraints may have compromised patients comfort. Future studies may need to incorporate more follow-up appointments to assess comfort levels better. Another limitation of the study was that the patient satisfaction questionnaire was only administered to patients who could understand and respond to questions in English. This restriction may have further impacted the sample size. Non-English-speaking patients were provided verbal explanations and asked questions in Malay and Tamil; however, this limitation still affected the overall representation of the patient population.

## Conclusion:

Our study shows that the majority of patients at Penang International Dental College were satisfied with their complete denture treatments, scoring from average to excellent across 10 parameters. No significant association was found between satisfaction and prior denture use or gender. Additionally, patients expressed high satisfaction with the operator's demeanour and performance.
